# Mono-ADP-ribosylation, a MARylationmultifaced modification of protein, DNA and RNA: characterizations, functions and mechanisms

**DOI:** 10.1038/s41420-024-01994-5

**Published:** 2024-05-11

**Authors:** Hao Wu, Anqi Lu, Jiuzhi Yuan, Yang Yu, Chongning Lv, Jincai Lu

**Affiliations:** 1 College of Traditional Chinese Materia Medica, Shenyang Pharmaceutical University, Shenyang, China; 2 Liaoning Provincial Key Laboratory of TCM Resources Conservation and Development, Shenyang Pharmaceutical University, Shenyang, China

**Keywords:** Molecular biology, Diseases

## Abstract

The functional alterations of proteins and nucleic acids mainly rely on their modifications. ADP-ribosylation is a NAD^+^-dependent modification of proteins and, in some cases, of nucleic acids. This modification is broadly categorized as Mono(ADP-ribosyl)ation (MARylation) or poly(ADP-ribosyl)ation (PARylation). MARylation catalyzed by mono(ADP-ribosyl) transferases (MARTs) is more common in cells and the number of MARTs is much larger than poly(ADP-ribosyl) transferases. Unlike PARylation is well-characterized, research on MARylation is at the starting stage. However, growing evidence demonstrate the cellular functions of MARylation, supporting its potential roles in human health and diseases. In this review, we outlined MARylation-associated proteins including MARTs, the ADP-ribosyl hydrolyses and ADP-ribose binding domains. We summarized up-to-date findings about MARylation onto newly identified substrates including protein, DNA and RNA, and focused on the functions of these reactions in pathophysiological conditions as well as speculated the potential mechanisms. Furthermore, new strategies of MARylation detection and the current state of MARTs inhibitors were discussed. We also provided an outlook for future study, aiming to revealing the unknown biological properties of MARylation and its relevant mechanisms, and establish a novel therapeutic perspective in human diseases.

## Facts

- MARTs catalyze MARylation, the hydrolases reverse MARylation and ADP-ribose binding domains recognize MARylation.
- The newly identified MARylated substrates includes protein, DNA and RNA, and possess physiological and pathological roles in mammal via diverse mechanisms.
- Targeting MARylation and MARylation-associated enzymes are promising therapeutic perspectives in human diseases.

## Open questions

- What are the biofunctions of nucleic acids MARylation, especially RNA MARylation.
- Is there any undiscovered enzymes regulating MARylation reactions.
- What is the potential connection between MARylation and PARylation.

## Introduction

**Fig. 1:**
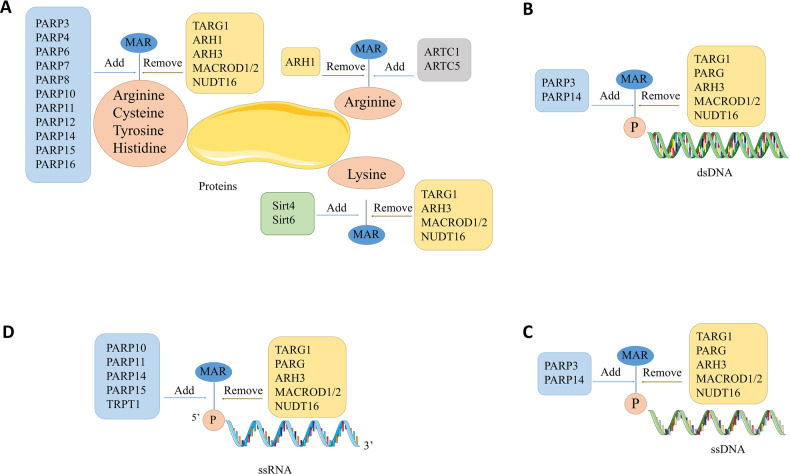
mono-ADP-ribosylation of protein and nucleic acids. **A** MARylation of proteins. PARP3, 4, 6, 7, 8, 10, 11, 12, 14, 15 and 16 MARylates acceptor amino acid residues including arginine, cysteine, tyrosine and histidine that cleaved by TARG1, ARH3, MacroD1/2 and NUDT16. **B** MARylation of dsDNA termini. PARP3, 14 MARylates phosphorylated groups of dsDNA ends that removed by TARG1, PARG, ARH3, MacroD1/2 and NUDT16. **C** MARylation of ssDNA termini. PARP3, 14 MARylates phosphorylated groups of ssDNA ends that hydrolyzed by PARG. **D** MARylation of ssRNA termini. PARP10, 11,14, 15 and TRPT1 MARylates 5’ phosphorylated ssRNA which reversed by TARG1, PARG, ARH3, MacroD1/2 and NUDT16.

ADP-ribosylation is a dynamic covalent modification that highly conserved throughout almost all domains of life [1]. The processes of this modification include transferring the ADP-ribose from nicotinamide adenine dinucleotide (NAD^+^) to its specific substrate meanwhile releasing nicotinamide. ADP-ribosylation was firstly defined as a post transcriptional modification (PTM) of protein in the sixties [2], followed by described crucial roles in the DNA damage responses [3]. In the following decades, major cellular processes involving cell growth and differentiation, transcription, stress responses, metabolisms and immunity were sequentially proved to be modulated by ADP-ribosylation as a PTM [4]. Of note, recent studies demonstrated ADP-ribosylation of nucleotides, besides protein targets [5]. In this enzymatic reaction, according to their roles, the enzymes can be categorized as ADP-ribosyl transferases (ARTs) which add ADP-ribose and ADP-ribosyl hydrolases which remove the ADP-ribose (Fig. 1). ADP-ribosylation is grouped into two patterns that MARylation identified by a single ADP-ribose unit linked to targets while PARylation containing polymers of ADP-ribose unit modification. Compared to the roles of PARylation and poly-ADP-ribosyl transferases, most notable PARP1, in cellular pathways are well-studied, the biofunctions of MARylation and MARTs are not fully elucidated, and MARylation-targeted therapeutics is still in the initial stage. However, knowledge of MARylation is rapidly developing and expanding. A growing number of evidence support MARylation is not only solely regarded as a type of PTM but also serving as a reversible modification targeting DNA and RNA. Furthermore, with the involvement of MARylation in essential cellular processes such as cancers, DNA repair, viral infection and cell cycle has been continuously reported, novel insights about MARylation attracts increasing attentions.

In this review, firstly, we provided an overview of MARTs catalyzing this novel modification, the hydrolases reversing it and ADP-ribose binding domains recognizing it. Secondly, we summarized the latest information about newly identified MARylated substrates including protein, DNA and RNA. And we emphasized on the physiological and pathological roles of these reactions in mammal as well as revealed the underlying mechanisms. Thirdly, we discussed the current methods for ADP-ribosylation detections and the state of MARTs inhibitors. Finally, we provided an outlook for future study, aiming to further analyzing the unclear biological functions of MARylation and their relevant mechanisms, and providing novel therapeutic perspectives in human diseases via targeting MARylation-related enzymes.

## ADP-ribosyl transferases

The ARTs are defined by their catalytic domain, a polypeptide fold binding NAD^+^ and transferring ADP-ribose onto substrates covalently. There are 22 known human ARTs which grouped into two classes based on key amino acids in their catalytic domain. The ARTs containing a histidine-tyrosine-glutamate triad (H-Y-E motif) are grouped into ADP-ribosyl transferase diphtheria-toxin like (ARTDs) [6]. While the ARTs containing an arginine-serine-glutamate sequence (R-S-E motif) are referred to ADP-ribosyl transferase cholera-toxin like (ARTCs), also called ecto-ARTs [7]. In addition to these two ARTs families, two Sirtuins members are also confirmed to possess ART-like catalytic activity. Based on the number of ADP-ribose units, ADP-ribosylation could be in the form of a single ADP-ribose unit catalyzed by MARTs or polymers of ADP-ribose units modified by poly(ADP-ribosyl) transferases (Table 1).

**Table 1:** Summary of human ARTs.

Name	Alternative name	Catalytic activity	Catalytic sequence	Substrates [4, 9]	Localization [196, 197]
PARP1	ARTD1	PAR	H-Y-E	Proteins, DNA	Nuclear
PARP2	ARTD2	PAR	H-Y-E	Proteins, DNA	Nuclear
PARP3	ARTD3	MAR	H-Y-E	Proteins, DNA	Nuclear
PARP4	ARTD4	MAR	H-Y-E	Proteins	Cytosolic (Vault particles)
PARP5a	TNKS1, ARTD5	PAR	H-Y-E	Proteins	Cytosolic (Stress granules)
PARP5b	TNKS2, ARTD6	PAR	H-Y-E	Proteins	Cytosolic
PARP6	ARTD17	MAR	H-Y-I	Proteins	Cytosolic
PARP7	tiPARP, ARTD14	MAR	H-Y-I	Proteins	Nuclear, Cytosolic (Microtubule)
PARP8	ARTD16	MAR	H-Y-I	Proteins	Cytosolic
PARP9	BAL1, ARTD9	MAR	Q-Y-T	Proteins	Nuclear, Cytosolic
PARP10	ARTD10	MAR	H-Y-I	Proteins, RNA	Nuclear, Cytosolic
PARP11	ARTD11	MAR	H-Y-I	Proteins, RNA	Cytosolic
PARP12	ARTD12	MAR	H-Y-I	Proteins,	Cytosolic (Golgi, SGs)
PARP13	ZAP, ARTD13	Inactive	Y-Y-V		Cytosolic
PARP14	BAL2, ARTD8	MAR	H-Y-L	Proteins, DNA	Nuclear, Cytosolic (SGs)
PARP15	BAL3, ARTD7	MAR	H-Y-L	Proteins, RNA	Cytosolic (SGs)
PARP16	ARTD15	MAR	H-Y-Y	Proteins	Cytosolic (ER, Ribosome)
ARTC1	ecto-ART1	MAR	R-S-E	Proteins	Cell membrane
ARTC3	ecto-ART1	Inactive	K-L-V		Cell membrane
ARTC4	ecto-ART1	Inactive	G-S-E		Cell membrane
ARTC5	ecto-ART1	MAR	R-S-E	Proteins	Secreted enzyme
Sirt4		MAR		protein	Cytosolic (mitochondria)
Sirt6		MAR		protein	Nuclear

### ARTDs

ARTD family include 17 poly (ADP-ribose) polymerases (PARPs) and a divergent PARP homolog tRNA2 -phosphotransferase (TRPT1) [8–10]. Some members generate PAR and others are limited to MARylation. Actually, there are only four ARTD members including PARP1, PARP2, and Tankyrases (PARP5a and PARP5b) are able to synthetize long PAR chains that leading to PARylation. In contrast, majority ARTDs are MARTs which transfer only a single ADP-ribose unit resulting in targets MARylation. These enzymes are PARP3, PARP4, PARP6-12 and PARP14-16 which sharing a common characterization that no glutamate catalytic activity [11]. PARP9 and PARP13 are both regarded as no catalytic activity. During MARylating reaction, cleaving the covalent linkage between a ribose and a nicotinamide molecule is the rate-limiting process, and subsequently, the single ribose is to be connected to targets. The ARTDs possess diverse cellular distribution [12] and modify various substrates. Protein was first identified as an acceptor of ADP-ribosylation and most enzymes are known to be protein-modifying. However, some of others are described to modify DNA, RNA or chemical groups for example phosphate.

### ARTCs

There are four ARTCs members in human [7]. These four ARTCs consist of two active members (ARTC1, ARTC5) and two inactive members (ARTC3, ARTC4). Since lacking the R-S-E motif, ARTC3 and ARTC4 cannot interact with NAD^+^. In despite of different binding mechanisms, ARTCs possess the similar configuration of NAD^+^ within the binding pocket as ARTDs. To date, protein is the only identified target of ARTCs-mediated MARylation and all active ARTCs conduct MARylation specifically onto arginine residues. ARTC1, 3 and 4 are localized in cellular membrane while ARTC4 is a secreted protein.

### Sirtuins

Sirtuins is a class of deacetylases group with seven enzymes in human. Sirt4 and Sirt6 possess MARyation activity in addition to two above ART superfamilies [13]. Sirtuins cleave acyl groups in NAD^+^-dependent manner and generate O-acyl-ADP-ribose (OAADPr) followed by transferred to target residues such as Lysine [14, 15]. Sirt4 is localized in cytoplasm, mainly mitochondria, while Sirt6 is in nucleus.

Substantially, although current evidence presented the knowledge about ARTs, some of them are yet poorly understood and their structures and modification sites are required to identify. In addition, the modification and conjunction sites of ARTs, in particular, MARTs are not well established that remain to be solved.

## ADP-ribosyl hydrolases

As a reversible modification, ADP-ribosylation is tightly regulated by three enzyme families including the DraG (dinitrogenase reductase activating glycohydrolase)-like ADP-ribosyl hydrolase family (ARHs), macrodomain-containing family and the nucleoside diphosphate linked to a variable moiety X (Nudix) family (Table 2). Additionally, an ADP-ribosyl hydrolase named NADAR was very recently found in bacteria and most of the eurkaryotes [16]. Due to no obvious homologues of the NADAR enzyme in humans, we do not further discuss NADAR in this article.

**Table 2:** Summary of ADP-ribosyl hydrolases.

Group	Name	Catalytic activity	Targets	Localization	Functions [42, 198]
ARHs family	ARH1	De-MAR,	Protein,	Cytosol	Bacterial infection, Cancer progression
	ARH2	Inactive		Cytosol	
	ARH3	De-MAR, De-PAR, De-OAADPr	Protein, dsDNA, ssRNA	Nucleus, Cytosol (mitochondria)	Genome stability, Parthanatos regulation, Antioxidant
Macrodomain-containing family	PARG	De-MAR, De-PAR	Protein, dsDNA, ssDNA, ssRNA	Nucleus, Cytosol	DNA repair
	MacroD1	De-MAR, De-OAADPr	dsDNA, ssRNA	Cytosol (mitochondrial)	DNA repair, cellular senescence
	MacroD2	De-MAR, De-OAADPr	dsDNA, ssRNA	Cytosol	DNA repair, nervous disorder
	TARG1	De-MAR, De-OAADPr	dsDNA, ssRNA	Nucleus	Insulin sensitivity
Nudix family	NUDT9	De-PAR	Protein	Cytosol (mitochondrial)	Energy metabolism, Calcium homeostasis
	NUDT16	De-MAR De-PAR De-OAADPr	Protein, dsDNA, ssRNA	Nucleus, Cytosol	DNA damage repair

### ARHs family

The ARH family contains three enzymes (ARH1, ARH2, and ARH3) with structurally similar catalytic domains including 290-360 residues, especially, ARH1 and ARH2 sharing a higher degree of sequence homology [17, 18]. ARH1, the first described ARH enzyme, specifically reverses MARylated arginine [19]. ARH1 is able to hydrolyze the *N*-glycosidic bond between a single ADP-ribose and arginine [20], while has weak capacity against PAR and OAADPr. Given that O-glycosidic is the main linkage between ADP-ribose and nucleic acids, ARH1 is not able to remove ADP-ribose from DNA or RNA [5]. The function of ARH1 is poorly understood despite a study showed that ARH1 deficiency promoted persistence of ADP-ribosylation and resulted in more sensitivity to Vibrio cholera infections in mice [21]. Due to lack of an aspartate in the catalytic center, ARH2 appears to be catalytically inactive [5]. ARH3 plays important roles in neurodegenerative disease [22] and efficiently uses PAR chains, MARylated serine and OAADPr as a substrate. ARH3 is the only hydrolase which able to cleave serine-linked mono(ADP-ribose) [23]. According to its specificity, ARH3 removes ADP-ribose from protein, DNA and RNA [23–25]. ARH3 is localized in both nucleus and mitochondria [18]. Nucleus-localization is responsible for its role in DNA damage [26] and regulation of parthanatos, a special type of cell death [27]. Mitochondria-localization of ARH3 may participate in the degradation of OAADPr as a metabolite in Sirtuins-mediated deacetylation reaction [28].

### Macrodomain-containing family

Macrodomain-containing enzymes are widely distributed in all domains of life, and share a highly conserved ADP-ribose binding domain, known as macrodomain [29]. Macrodomains consist of 150–210 amino acids with the core motif harboring a three-layer sandwich architecture and are able to bind some OAADPr, MARylated, and PARylated proteins [30]. The poly-ADP-ribosyl glycohydrolase (PARG) is the only known members with PAR-hydrolyzing property but unable to remove the terminal ADP-ribose linked to substrates [31]. Notably, PARG is also able to reverse MARylation onto the 5’ and 3’ phosphorylated ssDNA and ssRNA [24, 25]. The functions of PARG are involved in DNA repair, replication forks and recovery from persistent replication stress [32]. Therefore, targeting PARG is regard as a novel approach for modern chemotherapy [33]. Additionally, The mutation of PARG is clearly associated with neurodegeneration and accumulation of PAR in the CNS [34]. There are also three other human macrodomain-containing family members which have hydrolytic activity towards MARylated substrates: MacroD1, MacroD2 and TARG1. These enzymes were demonstrated to remove the single ADP-ribose unit from modified protein substrates instead of PAR chain. TARG1 also cleaves the ester linkage between glutamate-linked PAR although its activity is much lower compared with PARG [35]. Importantly, MarcoD1, MacroD2 and TARG1 can cleave the single ADP-ribose from the 5’ or 3’ terminal phosphates of dsDNA and ssRNA to reverse nucleic acids modification [24, 25]. The roles of MacroD1, MacroD2, and TARG1 in normal cell biological processes are not well understood. A few studies have revealed the biofunctions of these macrodomain-containing enzymes. Deficiency of TARG1 causes neurodegenerative disorder in an autosomal recessive inheritance pattern [35]. TARG1 could also be related to insulin sensitivity. MACROD1, a mitochondrial enzyme, overexpressed in various tumor tissues is proposed to participate in growth and invasion of cancer cells [36, 37]. As, so far, only be detected in the brain, the MACROD2 has been reported to correlated with autism-spectrum disorders [38, 39], which is supported by behavioral phenotypes observed in *MACROD2* gene knockout mice model [40].

### Nudix family

The Nudix family, a class of pyrophosphatases, has been found to participate in metabolism of ADP-ribose. Instead of completely removing the ADP-ribose moiety, the Nudix digest the phosphodiester bond which links the adenosine to the ribose moiety in ADP-ribose, liberating the AMP or phosphoribose AMP. In Nudix family, only NUDT9 and NUDT16 have been shown to hydrolyze protein linked ADP-ribose [41, 42]. NUDT9 possesses phosphodiesterase activity against PARylated substrates and OAADPr while its hydrolytic capacity is much lower than that of NUDT16 [41, 43]. NUDT16 is the only Nudix member which digest both MARylated and PARylated substrates including PARylated protein and DNA, and MARylated protein, DNA and RNA, as well as OAADPr [44]. Notably, Ectonucleotide Pyrophosphatase/Phosphodiesterase 1 (ENPP1), a type II transmembrane glycoprotein, possesses equivalent activity to NUDT16 on ADP-ribosylated proteins. As reported, ENPP1 is able to convert protein-linked MAR and PAR into ribose-5′-phosphate [45].

In general, ADP-ribosyl hydrolases inversing the ADP-ribose modification functions as key regulators of this signaling. But yet, little is known that how these hydrolyses are modulated for example coordination with undefined cofactors. Furthermore, the binding sites’ specificity and the linkage selectivity are needed further detailed elaboration. Given that, better clarify molecular interactions between ADP-ribosylation hydrolyses and ARTs will contribute to revealing the mechanisms in charge of ADP-ribosylation reversal. The physiological and pathological functions associated with these enzymes remains largely unclear and further studies are required to clarify these issues.

## ADP-ribose binding domains

In order to mediating downstream events, it is essential for ADP-ribosylation tags to be recognized by ADP-ribose binding proteins to read this modification. A number of studies have discovered different motifs, domains, or modules in proteins interacting with various forms of ADP-ribosylation.

### PAR-binding motifs

The PAR-binding motif (PBM), a short 20 hydrophobic amino acid motif, is the most common PAR-reader domain. PBM’s directly bind to ADP-ribose likely based on electrostatic interactions between the positively charged amino acids in the PBM and the negatively charged PAR chains [46]. Although the exact nature of its interaction with PAR remains unclear, PBM is found in hundreds of proteins which involved in DNA-related processes, RNA regulation, cell-cycle regulation as well as apoptosis and parthanatos [47–49]. For instance, hexokinase, the rate-limiting enzyme of glycolysis, contains a strong PBM [50]. PARylation of hexokinase inhibits its catalytic activity and induces bioenergetic collapse in parthanatos [50]. Interestingly, multiple PBMs can simultaneously appear in a same protein and may cooperate in their interaction with PAR chains by increasing affinity and specificity [46, 47].

### Macrodomains

The macrodomain, a sizable and globular module containing 130-190 amino acids, has been investigated the most extensively among the widespread ADP-ribose-binding domains. Macrodomains can interact with mono-ADP-ribose or the terminal ADP-ribose unit in poly-ADP-ribose chains and is responsible for reading variety of MARylated and/or PARylated substrates including proteins, free ADP-ribose, nucleic acids and ADPr metabolites [51]. Macrodomains participate in diverse biological events such as DNA repair, cellular signal transduction, transcription, genomic stability, immune response, different types of cell death [52]. Macrodomains are found in diverse proteins. Macrodomains act as readers of ADP-ribosylation in some macrodomain-containing proteins including macroPARPs (PARP9, PARP14 and PARP15), macroH2A1.1 and the chromatin remodeler ALC1 [53–56]. In contrast, macrodomains act as erasers of ADP-ribosylation in other macrodomain-containing proteins including ADP-ribose hydrolases (PARG, TARG1, MacroD1 and MacroD2) [29, 35, 51]. Interestingly, PARP14 is the only PARP with both transferase and hydrolase activities in one polypeptide due to it possessing both hydrolytic and binding macrodomains [57]. PARP14 exhibit ADP-ribosylhydrolase activity on protein and nucleic acid substrates [57].

### WWE domains

The WWE domain is named after its three most conserved amino acids (tryptophan-tryptophan-glutamate). Unlike macrodomains, the WWE domains commonly prefer to recognize PARylated targets due to its pocket binds to the *iso*-ADP-ribose (the base-ribose linker between two ADP-ribose units) rather than an ADP-ribose unit [58–60]. Four residues including Y107A, Y144A, R163A, and Q153A in WWE domains are crucial for the recognition capacity [61]. Interestingly, this domain is also present in some E3 ubiquitin ligases [61, 62], highlighting the crosstalk between ADP-ribosylation and ubiquitylation, and supported by recent reviews [63, 64]. The best-characterized PAR-targeted E3 ligase is RNF146 (also named Iduna) which catalyzing the ubiquitylation and degradation of PARyated protein substrate [65–69], and influences on diverse biological processes [70]. It is very interesting that in ART family, the members who carry WWE domains appear to be MARTs including PARP7 and PARP11-14.

### The PAR-binding zinc finger (PBZ)

PAR binding zinc fingers (PBZ) is a Cys2-His2 type zinc finger motif that binds PAR molecules contained in some DNA damage response proteins. The PBZ domain consists of a conserved motif with less than 30 amino acids and recognizes two adjacent ADPr units by hinging on adenine bases [71]. Similar to other zinc fingers, PBZ requires zinc for nanomolar affinity to its binding partner. Notably, compare to a single PBZ, the two zinc finger motifs cooperate to achieve high affinity for ADPr binding [72, 73]. Aprataxin polynucleotide kinase (PNK)-like factor (APLF) is a key regulator in DNA repair. Deletion of APLF in human cells leads to reduction of DNA repair rates following ionizing radiation [74]. APLF contains two tandem PBZ domains which are required for APLF recruitment to DNA damage sites [72].

### RNA and DNA binding motifs

One of the most intriguing recent fundings is that ADP-ribose can be recognized by protein motifs that are able to bind to RNA or DNA. Similar to nucleic acids, the ADP-ribose carries a negative charge because of its phosphate backbone. Because these macromolecules tend to bind positively charged amino acid residue, some competition exists among DNA, RNA or PAR for the same binding site.

### The RGG/RG motifs

The arginine*/*glycine-rich (RGG*/*RG) domain, also called glycine-arginine-rich (GAR) domains, is one of the most common RNA-binding domain throughout eukaryotes [75]. The RGG/RG motifs include RGG and RG repeats of varied lengths interspersed with spacers of different amino acids. Thousands of human proteins are reported to contain the RGG/RG motifs and involved in numerous cellular functions, such as DNA damage pathway, transcription, RNA processing, apoptosis and more [75, 76]. The RGG*/*RG domain binds to RNA in a non-specific sequence manner based on the interaction between its positively charged arginine residues and the negatively charged RNA chain [77]. Interestingly, The RGG/RG domain can also recognize PAR chain mediate downstream events. A number of studies demonstrated that some RNA-binding proteins containing the RGG/RG domain assembled in PAR-dependent way in response to genotoxic stress [78–81].

### The RRM

The RNA recognition motif (RRM) is an abundant RNA binding domains which targeting multiple RNA sequences and structures. Although with lower affinity than for their preferred substrate, RRMs also recognized PAR molecule as a nucleic acid-like sequence. Some RRM-containing proteins assemble at sites of PAR formation and promote genome stability [82–84]. Notably, there is a dynamical competition between RNA and PAR, and various RNA-binding proteins to be assembled at PARylated sites.

### The OB-fold

The oligonucleotiode*/*oligosaccharide binding fold (OB-fold), a 70–150 amino acid containing, is responsible for the interaction with single-stranded DNA (ssDNA) or oligosaccharides [85]. The exposed ssDNA is unstable and vulnerable to chemical attack and nucleolytic degradation, and needs to be properly protected to avoid mutations. Therefore, the OB-fold-containing proteins participate in multiple DNA metabolic processes including DNA replication, DNA repair, cell cycle regulation, and maintenance of telomeres [86]. Similar to the RGG/RG motifs, the OB-fold can also act as a PAR reader. Evidence supported that the OB-fold recognizes *iso*-ADP-ribose and bind to PAR chain [87, 88].

### PIN domains

PIN domains are named after the amino terminus of the PilT protein and contain approximately 130 amino acids in length. Proteins possessing PIN domains are present in all kingdoms of life and act in a metal-iron-dependent way, usually through magnesium or manganese ion [89]. The PIN domains are evolutionarily conserved and recognize single-stranded DNA or RNA. A certain number of studies also confirm that the PIN domains bind to PAR with relatively high affinity, and this interaction is required for the rapid recruitment of the related protein to DNA break sites in response to DNA damage [90].

### KR-rich and SR repeats motifs

Some of the most highly enriched PAR readers such as lysine- and arginine rich (KR-rich) motifs and Serine*/*Arginine repeats (SR repeats) do not contain a classic ADP-ribose binding domain. However, they have repeats of positively charged residues [91]. The positively charged arginine residues contribute to a strong electrostatic interaction with negatively charged PAR chains. Therefore, their affinity for both RNA and PAR is based on the positive charges associated with the arginine rich repeats.

### CCCH zinc finger domains

Zinc finger proteins are commonly regard as DNA-binding transcription factors. However, Cys–Cys–Cys–His (CCCH) zinc finger proteins are capable of binding to target RNA and regulate RNA metabolism such as mRNA splicing, polyadenylation, export, translation and so on. The CCCH zinc finger domains consist of three conserved cysteines followed by a histidine that coordinated with a zinc ion, maintaining the structural stability [92]. So far, over fifty CCCH zinc finger proteins have been found in in humans and mice. Increasing functions of CCCH zinc finger proteins are being revealed such as regulation of cell differentiation and tumor growth, cytokine production, immune cell activation and immune homeostasis [93, 94].

Collectively, several modules have been identified to recognize ADP-ribose and play pivotal roles in the transduction of ADP-ribose signals, however, it appears that only macrodomains can interact with MAR. Hence more domains functions as readers of MAR are still to be further discovered.

## MARylation of protein

PTM is a common tool to media a rapid change of the cellular status via adding chemical moieties. Protein was firstly identified as the substrate of ADP-ribosylation, subsequently various protein targets were discovered. As a highly conserved PTM, MARylation of proteins either directly alters protein activity or impacts on their interactions with other molecules. Unlike serine residues as the major acceptor sites for PARylation [95], the major MARTs are capable of modifying a variety of amino acid residues of modified proteins involving arginine, cysteine, tyrosine and histidine [96] in addition to serine [97]. Similar with other types of PTM, MARylation is also in a reversible pattern and the macrodomains are key modules that regulates MARylation.

## Functions of protein MARylation

Conjugation of mono-ADP-ribose to proteins plays crucial signaling functions in various biological processes including RNA biology, cancer, cell-cycle, DNA damage repair, inflammatory response, virus infection and so on (Table 3).

**Table 3:** Biological functions of proteins MARylation.

Function	MART	MARylated target	Ref
DNA damage	PARP3	NuMa, the histone H2B, PARP1, PARP3	[99–102, 199]
	PARP9	Dtx3L	[103]
	PARP10	PCNA	[107]
	PARP14	PCNA, MRE11	[108, 109]
	Sirt6	PARP1, KAP1	[15, 111]
Cancers	PARP6	Chk1 kinas	[112]
	PARP7	HIF-1α, c-MYC, estrogen receptor, androgen receptor, α-tubulin	[113–116]
	PARP9	Dtx3L, IRF1	[117, 118]
	PARP10	PLK1	[119]
	PARP12	FHL2	[120, 123]
	ARTC1	Integrin α7	[121, 122]
Virus infection	PARP7	TBK1	[126]
	PARP10	nsP2,	[131, 132]
	PARP11	β-transduction repeat-containing protein	[124]
	PARP12	NS1, NS2, GAPDH	[130, 133]
	PARP14	STAT1	[127]
	PARP9	N/D	[127]
Inflammation	PARP10	NEMO	[136]
	PARP14	STAT1	[127]
	PARP9	N/D	[127]
	ARTC1	HNP-1	[138]
Lipid metabolism	PARP7	LXR-α, LXR-β	[139]
	PARP10	N/D	[140, 141]
Cell-cycle	PARP10	Aurora-A, RAN	[142]
	ARTC1	PDGF-BB	[144]
RNA biology	PARP7	AHR	[147]
	PARP16	RPL24 and RPS6	[145]
	N/D	EF2	[146]
	PAR13	Ago2	[128]
ER stress	PARP16	PERK and IRE1α	[149]
	ARTC1	GRP78/BiP	[150]
Autophagy	PARP10	p62	[151, 152]
	PARP12	p62	[151, 152]
Insulin secretion	Sirt4	GDH	[154]

### DNA damage

The functions of PARylation in response to genotoxic stress have been so far well-established. However, recent studies provide novel insights into roles of MARylation in DNA damage repair. PARP3 is a DNA-dependent PARP and specific functions in cellular response to DNA strand breaks. PARP3 is able to be activated by DNA nicks, breaks and gaps possessing 5′ phosphorylated ends. The non-homologous end joining (NHEJ) is a classical double-strand breaks repair pathway in two DNA termini are directly ligated and active throughout whole cell cycle. PARP3 is shown to be efficiently recruited to DNA damage sites and to interact with different substrates belonging to the NHEJ pathway [98]. Moreover, PARP3 regulate mitotic progression and DNA damage repair by modifying the mitotic spindle components NuMa and the histone H2B, respectively [99, 100]. PARP3 also binds to PARP1 and activates PARP1, even in the absence of DNA, leading to synthesis of PAR chain [101]. Interestingly, PARP3 even modifies itself following their DNA-dependent activation [102]. PARP9 combines with E3 ligase Dtx3L to form heterodimer that is localized DNA damage sites and contributes to DNA repair response [103, 104]. Interesting, PARP9/DTX3L complex makes a hydbrid MAR-ubiquitin modification which also supports the connection between ubiquitination and ADP-ribosylation [105]. PARP10 has been the first identified MARylating enzyme and possesses multiple functions based on modification of its protein substrates [106]. A study showed that PARP10 regulates DNA damage hypersensitivity via binding to proliferating cell nuclear antigen (PCNA), a machinery component of DNA replication [107]. Consistently, PARP10 knockdown lead to sensitivity enhancement to DNA damage. PARP14 can also bind with PCNA to promote DNA replication and genomic stability in human cell lines [108]. While the opposite outcomes were shown in BRCA-deficient cells upon replication stress, in this study, PARP14 interacted with MRE11, a nuclease for replication forks degradation, to form a complex based on its catalytic activity and subsequent promoted genomic instability and DNA injury hypersensitivity [109]. These contradictory outcomes appear to be due to the diverse functions of PARP14-specific protein targets, supporting multiple roles of MARylation in DNA damage regulation. Notably, although lack of direct experimental evidence, PARP4 actually contains a BRCT domain which responsible for interacting with DNA damage repair proteins [110], indicating the potential role of PARP4 in DNA repair. Additionally, Sirt6 is recruited to DNA break sites and improves DNA repair by activation of PARP1 in MARylation-dependent way, and this action also strongly indicates the potential crosstalk between MAR and PAR [15]. KRAB-associated protein 1 (KAP1), a nuclear corepressor protein, is the other modified target of Sirt6. SIRT6 MARylates KAP1 to promote gene stability in response to DNA damage [111]. Current research has expanded the understanding of MARTs in DNA repair, however, less is known about the rest ones upon this context and further study needs to address this.

### Cancers

The cancer-linked roles of MARylation and MARTs is relatively well-established. PARP6, a mono-ADP-ribose generating enzyme, directly MARylates Chk1 kinas which involved in regulating centrosome functions to promote tumorigenesis in breast cancer [112]. Correspondingly, PARP6 specific inhibitor AZ0108 induced the multipolar spindle phenotype to result in apoptosis of breast cancer cells [112]. In breast and colon cancer cells, PARP7 down-regulates oncogenic transcription factors such as HIF-1α, c-MYC, and estrogen receptor to modulate the War-burg effect and tumorigenesis. Mechanistically, PARP7 promotes these protein substrates ubiquitination and proteasomal degradation based on its MARylation activity [113]. Consistently, deletion of PARP7 in breast and colon cancer xenografts promotes tumorigenesis. This evidence also highlighted the crosstalk between MARylation and ubiquitination. In addition, PARP7 was reported to MARylate androgen receptor (AR) on its multiple cysteine residues to restrain AR-signaling in prostate cancer cells [114, 115]. Given the crucial role of AR prostate cancer, the PARP7-AR axis may be a potential therapeutic strategy for prostate cancer. However, in ovarian cancer, PARP7 plays the opposite role. As was reported, PARP7 MARylates a-tubulin, a cytoskeletal protein, to destabilize microtubules and facilitates tumor growth and motility, and PARP7 knockdown results in inhibition of tumor growth, migration and invasion [116]. These inconsistent data demonstrated the cancer-specific effects of PARP7. In general, MARylation mediated by PARP7 leads to a negative regulation of protein substrates and the multi-faceted effects probably due to the biofunctions of different targets and their downstream signaling. Notably, PARP9-Dtx3L complex not only regulates DNA repair but also exists in prostate cancer cells and possesses oncogenic capacity [117]. This complex inhibits interferon regulatory factor 1 (IRF1), a tumor suppressor, to promote the proliferation of different prostate cancer cell lines [117]. Similar in diffuse large B-cell lymphoma, PARP9 as a oncogenic co-factor represses the STAT1-IRF1 pathway in MARylation-dependent manner and upregulates two protooncogenes IRF2 and B-cell CLL/lymphoma (BCL)-6 levels [118]. Furthermore, PARP10 was reported to MARylate polo-like kinase 1 (PLK1), a kinase that relevant to tumor progression, to restrain its activity and oncogenic function in hepatocellular carcinoma [119]. The epithelial-mesenchymal transition (EMT) contributes to migration and invasion of tumor. PARP12 act as a tumor suppressor that controls hepatocellular carcinoma (HCC) metastasis via regulating the EMT process [120]. These novel functions occur through stabilization of the transcription factor FHL2, resulting in reduced levels of transforming growth factor beta 1 (TGF-β1), a EMT facilitator increasing mesenchymal marker levels and reducing epithelial marker levels. Integrin α7 is a cell surface adhesion molecule which regulates cell growth, migration and adhesion. MARylation of integrin α7 induced by ARCT1 modulate EMT and, accordingly, alteration of ARCT1 expression significantly changed the EMT markers levels [121, 122]. Given that poly-ADP-ribosyl transferases inhibitors as anticancer agents have successfully entered clinical practice [123], supporting the potential clinic uses of mono-ADP-ribosyl transferases inhibitors. Together, although strong evidence has supported the novel therapeutic strategies by targeting Mono-ADP-ribosyl transferases, further study still needs to identify the cancer-associated roles of MARylation and MAR-related enzymes.

### Virus infection

MARylation plays a complex role in antiviral response through modifying host and viral proteins. Interferons (IFNs), designated types FIN-I, FIN-II and FIN-III, mediate the rapid host response to viral infection and play key roles in the innate immune system. PARP11 MARylates the E3 ligase β-transduction repeat-containing protein to promote IFN-I receptor subunit 1 ubiquitination and degradation, leading to suppression of antiviral capacity [124]. Inhibiting PARP11 pharmacologically could stabilize IFN-I receptor subunit 1 and enhance IFN-I pathway transduction to improve the resistant of mice to viral infection [124]. PARP14 functions as the main antiviral PARP against coronavirus infection and plays a crucial role in the induction of interferon [125]. TBK1 is an important kinase that drives IFN-I generation. It was reported that PARP7 MARylated TBK1 to restrain its phosphatase activity and inhibited IFN-I-induced innate response against viral infection [126]. STAT1 functions as activator of IFN signaling. PARP14 induces MARylation of STAT1 to suppresses IFN signaling which reversed by PARP9 via interacting with PARP14 [127]. PARP12, an interferon-stimulated MARTs, localizes Golgi and is characterized by its anti-viral function. During stress stimulation, PARP12 reversibly translocate from the trans-Golgi network to stress granules triggered by PARP1 [128, 129]. NS1 and NS2 are both nonstructural proteins of Zika virus (ZIKV), and responsible for the host antibody response and viral immune evasion strategies. PARP12 possesses anti-ZIKV activity via initially MARylating NS1 and NS2 to induce their proteasome-mediated degradation in a PARP-dependent way [130]. Chikungunya virus (CHIKV) is a mosquito-borne virus and encodes a nonstructural polyprotein (nsP) followed by cleaved into four components (nsP1-nsP4). PARP10 has been demonstrated to MARylate nsP2 to prevent multiple protein processing and replication of CHIKV [131, 132]. Stress granules (SGs), localized in the cytoplasm, is closely related to viral infections and innate immunity response. PARP10 MARylates and recruits GAPDH into SGs without affecting its dehydrogenase activity [133]. Besides, PARP12, PARP13 and PARP15 were also localized in SGs [134], and PARP12, PARP14 and PARP15 were defined as components of heat shock-induced SGs [135], suggesting the potential roles of MARylation in SGs-linked stress responses. Collectively, targeting PARPs is a potential strategy for antiviral response and will become a research hotspot. So far, there are only a few substrates of MARylation that have been identified. Therefore, more relevant substrates from host and virus need to be defined.

### Inflammatory responses

Inflammation response is an essential immune reaction while uncontrolled injury will lead to a range of inflammation-related diseases. Nuclear factor kappa-light-chain-enhancer of activated B cells (NF-kB) is a nuclear transcription factor that plays a key role in inflammation and triggers transcription of pro-inflammatory cytokines such as interleukins (ILs), interferons (IFNs) and tumor necrosis factor (TNFs). PARP10 negatively modifies NF-kB signaling by MARylating NEMO, an important component of this pathway, upon the stimulation of IL-1β or TNF-a [136]. Macrophage activation is divided into pro-inflammatory and anti-inflammatory phenotypes. Pro-inflammatory macrophage activation participates in pathogenesis of inflammation-associated disorders. It was reported that PARP9 and PARP14 cross-regulated macrophage and possessed opposing functions in macrophages activation and arterial lesions [127]. Mechanistically, PARP14 MARylates Glu657 and Glu705 of signal transducer and activator of transcription 1 (STAT1) that implicated as a regulator of pro-inflammatory mediators to inhibit Try701 phosphorylation, and which suppressed by PARP9. These findings also suggest interplay between MARylation and phosphorylation in inflammatory diseases mediated by pro-inflammatory macrophage activation. In cancers, PARP14 plays an immune suppressive role via regulation of IL-4 and IFNs-γ signaling pathways [137]. RBN012759, a selective PARP14 inhibitor, reverses protumor gene expression in macrophages and mediates inflammatory response in human tumor explants [137]. Human neutrophil peptide 1 (HNP-1) is a crucial component of innate immunity response. ARTC1 MARylates HNP-1 on its arginine residue mediated multi-functions including inhibition of cytotoxic and anti-microbial roles while enhancement of IL-8 release and neutrophil recruitment [138].

### Lipid metabolism

Liver X receptors (LXRs) are nuclear receptors that responsible for the regulation of cholesterol, lipid, and glucose metabolism. It was reported that PARP7 could MARylate LXR-α and LXR-β subunits to enhance LXRs-dependent transactivation which inhibited by ADP-ribosyl hydrolase, MACROD1 [139]. As the downstream targets of LXRs, Sterol regulatory-element binding proteins (SREBPs) are responsible for lipid synthesis and PARP7 acts as a positive regulator of SREBP1 level via LXR pathway [139]. Downregulation of PARP10 level is associated with an increasing of fatty acid oxidation [140]. Furthermore. PARP10 knockdown results in a reduction of secreted apolipoprotein B, a main carrier of triglyceride-rich lipoproteins and low-density lipoprotein cholesterol [141].

### Cell cycle

The mitotic kinase Aurora-A is a critical player in centrosome function and mitosis. It was reported that PARP10 regulated the transition from the G2-phase to mitosis by MARylation of Aurora-A [142]. Depletion of PARP10 affects Aurora-A recruitment in centrosomes and exhibited a delayed progression in the G2/M phase. RAN is a GTPase and responsible for nucleocytoplasmic trafficking and mediation of mitosis [143]. RAN was identified as a MARylated substrates of PARP10 that partially revealing the mechanisms by which PARP10 regulates cell-cycle [54]. Moreover, ARTC1 can regulate mitosis though MARylating platelet-derived growth factor-BB (PDGF-BB) in human cell line [144].

### RNA biology

MARylation of RNA-regulatory proteins can affect their function and regulate RNA biology such as translation, transcription. Ribosomes are the key components participating in mRNA translation. A recent research showed that PAPP16-mediated MARylation of ribosomal proteins including RPL24 and RPS6 significantly restrained polysome assembly, mRNA loading and translation [145]. Elongation factor 2 (EF2) is a GTPase that participates in ribosome translocation. MARylation of EF2 suppressed its activity and inhibited protein synthesis even though the mechanism of this inhibitory effect is unknown [146]. Argonaute (Ago) proteins are other important ingredients of the RNA-induced silencing complex (RISC) which regulates mRNAs translation. PARP13 functioned in these processes via modifying Ago2 to induce translational repression [128]. In transcriptional aspect, it was found that PARP7 played an important role in transcription regulation via MARylating aryl hydrocarbon receptor (AHR) a ligand-activated transcription factor acting as a negative feedback regulator of transcription [147], and the PARP7-induced effects can be reversed by mono-ADP-ribosylase MACROD1 [148].

### Endoplasmic reticulum (ER) stress

Protein kinase RNA-like ER kinase (PERK) and inositol-requiring enzyme 1 (IRE1α) are two unfolded protein response-associated kinases that play key roles in ER stress. And a research demonstrated PERK and IRE1α were the substrates of PARP16, an ER transmembrane protein [149]. As reported, PARP16-induced MARylation of PERK and IRE1α increased their kinase activity and promoted downstream events [149]. Knockdown of PARP16 made cells more sensitive to ER stress and reduced cell viability. GRP78/BiP is a key ER chaperone and also an identified target of ARTC1. ARTC1 inhibited GRP78/BiP activity in MARylation-dependent way and led to reduction of protein synthesis and protein flux into ER in response to ER stress [150].

### Other new functions

MARylation also play roles in other physiological and pathological processes including autophagy, insulin secretion and so on. Some studies demonstrated that PARP10 and PARP12 could both interact with p62, a ubiquitin receptor associated with autophagy [151, 152], suggesting the their potential role of in autophagy. However, it is still lacking sufficient evidence to prove these functions are based on MARylation of their substrate p62. In addition, PARP10-mediated MARylation can directly regulate the kinase activity of glycogen synthase kinase 3β (GSK3β), a well-known enzyme playing important role in metabolism, immunity, cell proliferation and tumorigenesis [153]. Glutamate dehydrogenase (GDH) is documented to enhance glutamate and glutamine metabolism to promote insulin secretion. Sirt4 represses GDH activity by MARylation to reduce insulin release in response to glucose and amino acids [154].

## MARylation of DNA

ADP-ribosylation of DNA was first reported in 2001 [155]. This study showed that pierisin-1, an ARTC enzyme from the cabbage butterfly, is able to MARylate double-stranded DNA (dsDNA) at the N2 position of guanine [155]. Notably, the pierisin-1-mediated modification is irreversible. In 2021, tightly regulated reversible DNA ADP-ribosylation systems were reported in a variety of bacteria that control for instance DNA replication and growth [156]. In Mammalian, there are three DNA-associated PARPs including PAPR1-3 which commonly harbor a tryptophan-glycine-arginine (WGR) domain responsible for DNA binding. PARP1 and PARP2 are able to PARylate DNA to generate PAR-DNA adducts. However, among these three enzymes, PARP3 is the only DNA MARTs which catalyzes attachment of a single ADP-ribose onto phosphorylated DNA ends. PARP3 shows MARylation activity on both 3′ - and 5′ -terminal phosphorylated groups at double- and single-strand DNA (ssDNA) break ends, whereby the 5’-termini presents the preferred target [25, 157]. An in vitro experiment also demonstrated that PARP3 catalyzed MARylation at the 5′-phosphate terminal of gapped DNA, which can serve as a substrate of DNA ligases for ligation with dsDNA and also serve as primer for PARP1- or PARP2-induced PARylation [158]. Just recently, two studies showed that PARP14 could also MARylates both ssDNA with a phosphate group at the termini and nascent dsDNA [159]. Of note, MARylation of DNA ends is a reversible process. Several cellular known hydrolases such as PARG, MACROD2, TARG1 and ARH3 can remove the mono-ADP-ribose moiety covalently attached to DNA, and PARG is most efficient in removing ADP-ribosylation on DNA, supporting that DNA termini MARylation serve as a transient mark [25, 157]. Currently, (patho)physiological roles of DNA ADP-ribosylation in eukaryotes are not clear yet but there are many suggestions that it exists.

## Functions of DNA MARylation

PARP3 is stimulated by DNA double-strand breaks (DSBs) and associated with the cellular response to DNA damage. Co-immunoprecipitation studies of PARP3 identified various proteins that are involved in NHEJ DNA damage repair to induce the direct re-ligation of DNA lesions [98, 160]. In accord, punished data support the function of PARP3-dependent MARylation in this type of DNA damage repair [161, 162]. Given that PAR-DNA complex breakage sites are reported to be prevented from degradation by nucleases [163], together with the initial single ADP-ribose added by PARP3 can be extended by PARP1 and PARP2, it is reasonable to propose PARP3-mediated DNA MARylation may play a protective role by shielding breakage sites until repair proteins fully recruited during DNA damage. The involvement of PARP3 in chromatin compaction regulation and chromosomal rearrangements in cells also supports its participation in the DNA repair mechanisms [164, 165]. In opposite, PARP14 bound with nascent DNA to promote MRE11-mediated fork degradation and induced gene instability, as above mentioned. For now, MARylation of DNA ends likely functions as a modification for protection of targeted DNA from nucleases-mediated uncontrolled degradation and a signal for recruitment of DNA repair-associated proteins. However, the biochemical characterization of ADP-ribosylation at DNA breakage sites is still largely unclear and the function of this novel DNA modification in cells remains to be unraveled.

## MARylation of RNA

RNA has so far been demonstrated to serve as a substrate for MARylation catalyzed by MARTs involving PARP10, PARP11, PARP12, PARP14, PARP15 and a PARP like enzyme -TRPT1 [159, 166]. Some other MARTs such as PARP7 and PARP12 have been predicted to bind phosphorylated RNA ends [167]. PARP7, PARP12 and PARP13 contain zinc finger domains of the CCCH-type while PARP10 and PARP14 contain multiple RRMs. However, it was reported the catalytic domains of PARP4, PARP6, PARP13 and PARP14 as well as full-length PARP3 and PARP16 had no activity on the modification of 5’-phosphorylated RNA [24]. In contrary, a recent research proved evidence for first time single-stranded RNA (ssRNA)-binding ability and RNA MARylation capacity of both PARP14 and its fragment containing a KH domain which functions as a sequence-specific RNA-binding module [159]. PARP14 is not able to modify RNA substrates with unmodified ends but induce MARylation on a phosphorylated termini [159]. PARP10 can also MARylate phosphorylated ssRNA ends with a preference for 5′-terminal phosphate rather than 3’-terminal phosphate [24]. However, PARP10 is not able to modify ssDNA. Interestingly, the full-length PARP10 mortifies RNA ends with less efficient activity than its catalytic domain polypeptide alone that probably due to an auto-inhibitory pattern involving an interaction of N-terminal sequences with the catalytic domain. In addition, PARP11 and PARP15 are also capable of catalyzing phosphorylated ssRNA. TRPT1, known for its essential function in the fungal tRNA splicing pathway, transfers ADP-ribose moiety to 5’-phosphorylated ends of RNA to form a 5’-cap structure, independent of oligomer length [24]. Importantly, a recent study presented that RNA MARylation occurs in human cells and is further confirmed to be conducted by TRPT1, PARP10, PARP11, PARP12, and PARP15 [166]. Similar to MARylated DNA, this non-canonical modification of RNA in cells can be reversed by cellular hydrolases including TARG1, PARG, ARH3, and MacroD1/2 indicating high dynamicity of this modification [24, 166]. Interestingly, a very recent study provides evidence that ADP-ribosylated RNA can be further ubiquitylated to produce a potential dual ubiquitin-ADP-ribose- modification [168].

## Functions of RNA MARylation

RNA functions are heavily dependent on post-transcriptional modifications. Before translated and degraded, pre-mRNAs have to undergo multiple processing processes involving 5′ capping, splicing, 3′ polyadenylation, and then generate mature mRNAs. To data, little is known about the role of RNA MARylation as a newly identified post-transcriptional regulator. Recently, a study in mammalian cells demonstrated that various cellular stressors such as IFN, MG132, Arsenite and H_2_O_2_ could regulate the level of MARylated RNA [166]. Furthermore, MAR-capped target mRNA is prevented from XRN1-induced degradation but not allowed by translation [166]. This exciting evidence indicates a potential mechanism that MAR acts as a novel mRNA cap and protects modified mRNA from degradation in cellular stress conditions, and then this modification is removed by ADP-ribosyl hydrolases until get rid of cellular stress. Two recent studies showed that PARP14 binds to cyclin D1 mRNA 3′UTR to regulate cell-cycle progression and interacts with tissue factor (TF) mRNA 3’UTR to regulate its mRNA degradation [169, 170]. Given that PARP14 functions as a RNA binding protein and possesses MARTs activity, a possibility is that PARP14 selectively MARylates mRNA 3′UTR in order to affect mRNA stability. Similarly, PARP13, a catalytically inactive enzyme, binds to a region in cellular TRAILR4 3’UTR to increase cell sensitivity to TRAIL-mediated apoptosis [171]. Moreover, PARP16, function as a RNA binding protein, modulates the mRNA stability of amyloid precursor protein (APP), the precursor of beta-amyloid (Aβ) and prevents modified APP from degradation in Alzheimer’s disease (AD) mice [172]. Notably, different from other MARylated mRNAs with significant inhibition of their translation in cellular stresses, PARP16-mediated modification promotes mRNA stability and increased APP protein level [172]. It is probably due to the chronic and degenerative pathological processes of AD, compared to acute stress conditions, and the neuronal damage role of PARP16 in AD which exacerbates APP production. Additionally, a recent study showed that PARP12 could interact with SARS-CoV-2 RNA and depletion of PARP12 led to viral SARS-CoV-2 RNA levels increased, indicating the antiviral function of RNA MARylation [173]. Generally, MARylation provides a novel potential mechanism for the post-transcriptional regulation of target gene expression, especially in response to cellular stresses. In this process, different MARTs catalyze different sites such as 3′UTR or 5′UTR. The main biological effects triggered by this modification are prevention of MARylated mRNA from degradation which is similar with that of DNA, and inhibition of target gene translation during acute cellular stresses. Given the identification of RNA MARylation is rather recent, it is still lack of sufficient in vivo data about RNA MARylation. Future studies in cells are necessary to sufficiently reveal RNA MARylation’s roles and impactions such as mRNA stability, localization and translation. It would also be interesting to identify specific MAR-capped RNAs, such as mRNA, transfer RNA and ribosomal RNA and so on.

## Detection of ADP-ribosylated substrates

Currently, techniques for MARylated substrates detection, especially endogenous MARylation are scarce, as a result, its regulation and specific sites remain largely unclear. Based on the ADP-ribosylation reaction, cofactor radiolabelled NAD^+^ as substrate is used to identify ADP-ribosylated substrates such as protein and nucleic acids. This method has high sensitivity and no effects on enzyme catalysis [24]. In addition, biotin-labelled NAD^+^ and NAD^+^ analogues have also used to label MARylated targets [153]. However, these modified NAD^+^ methods are limited to in vitro assays. In mammalian cells experiments, the first MAR-specific rabbit polyclonal antibody was produced in 1992 and targeted MARylated *Sulfolobus* *acidocaldarius* eEF2, an earliest known protein substrate of this modification [174]. Functional domains that bind to ADP-ribose have been used as alternatives to antibodies for ADP-ribosylation detection [175]. For example, Af1521 macrodomain interacts with MARylated and PARylated substrates while macro2*/*macro3 macrodomain prefer to recognize MARylated proteins [54, 176]. Encouragingly, the first protein site-specific antibodies that have very recently gotten available [177]. Furthermore, the toolkit for detection of ADP-ribosylation on DNA has been established in human cells [178]. Some groups have also developed agents with high affinity and specificity for MARylated RNA [24, 179]. Additionally, recent labeling methods including a clickable aminooxy alkyne (AO-alkyne) probe, enzymatic labeling of terminal ADP-ribose (ELTA) and N6 -propargyl adenosine (N6pA) tag provide a suite of techniques for ADP-ribosylation detection, although not always specifically targeting MARylation [180–183].

## Progress and challenges of MARTs inhibitors

So far, therapeutic benefits of PARPs inhibitors mainly have come from inhibitors of PARP1 and other nucleus PAPRs for cancer treatment [184]. However, selective inhibitors targeting MARTs are very limited. Recent advances in the understanding of MARTs structures and bioactivities lead to some progress in development of MARTs inhibitors. PARP3 modulates mitotic progression and DNA damage repair as mentioned above. A specific inhibitor of PARP3 known as ME0328 is in early-phase development, and has been proved to promote the chemotherapy agent vinorelbine cytotoxicity [185, 186], supporting the potential combination strategies of MARTs inhibitor with other therapeutic modalities. PARP7 also play key roles in several types of cancers. RBN-2397, the inhibitor specific for PARP7, can enhance type I IFN signaling to induce antitumor immunity dependent on CD8 T cells [187]. Different from nucleus PARPs inhibitors commonly tested as combination partners for chemotherapies [188], RBN-2397 administered alone represents complete and durable inhibition of tumor growth [187]. Now RBN-2397 is in Phase I clinical trials for solid tumors treatment (https://clinicaltrials.gov/ct2/show/NCT04053673). In 2016, Venkannagari et al identified OUL35 as a selective and potent inhibitor for PARP10. OUL35 reverses PARP10-induced human cell death and sensitizes cells to DNA damage [189]. In the follow-up study, Holechek et al designs PARP10/PARP14 selective inhibitors based on OUL35 that possess good metabolic stability against mouse liver microsomes [190]. Kirby group identifies a potent and selective PARP11 inhibitor ITK7 [191]. This study demonstrates that ITK7 inhibits PARP11 auto-MARylation and causes PARP11 to dissociate from the nuclear envelope [191]. Mariko et al. perform high-throughput screening and an immunoradiometric assay to identify PAPR14 inhibitors via competing for binding with NAD^+^ [192]. In 2021, the first highly potent and selective inhibitor of PARP14 RBN012759 has been reported. RBN012759 enables to reverse IL-4-driven protumor gene expression in macrophages and induces an inflammatory gene signature in kidney cancer tumor explants [137]. Subsequently, evidence shows that RBN012759 reduce stress granule assembly in ovarian cancer cells [193]. Wang et al characterizes epigallocatechin-3-gallate (EGCG), a major flavonoid of green tea, as a potential inhibitor of PARP16 [194]. Given that PARP16 play an important role in ER stress, EGCG has been shown to promote the ER stress-mediated cancer cells apoptosis [194].

In general, the development of clinical inhibitors of the known MARTs is still lagged behind. The efficacies of some inhibitors are not well-documented, and data about the safety and resistance of MARTs inhibitors are almost blank. Further research needs to evaluate their characteristics in vivo and even in patients. Moreover, current inhibitors of PARPs are mainly used for cancer therapy due to their roles in DNA repair pathway. Given the multiple functions of PARPs and MARTs in various diseases, MARTs inhibitors may also have potential effects on non-oncological indications such as inflammation, viral infection, lipid metabolic diseases. Notably, better screening techniques, reliable biomarkers of response and refined structural information will also transfer benefits to design and discovery of novel MARTs inhibitors. Addition to MARTs, the ADP-ribosyl hydrolases that remove MAR could also serve as targets for drugs development.

## Future perspectives

MARylation is a regulatory modification on protein or nucleic acids, and participates in modulation of various cellular pathways and plays crucial roles in health condition and multiple diseases. However, it is still in the initial stage and remains largely unknown. Therefore, there are several important issues that are required to be further addressed.

### To well understand biological activities and mechanisms

Compare to the functions of PARylation is well documented, we do not yet well understand the roles of MARylation in pathogenic and physiological conditions. Further studies are needed to address the roles of MARylation and MARylation-associated enzymes such as MARTs and MAR hydrolases in health and disease. At the same time, as a regulatory modification, the relevant molecular and biochemical mechanisms of MARylation on protein and nucleic acids, the mechanisms underlying their functions are also required to revealed. Furthermore, identification of novel MARylated target will contribute to better understanding of this modification. With the list of substrates growing, MARylation would become a most exciting cellular function.

### To increase in vivo tests

So far, most data about MARylation and MARTs derive from in vitro tests by commonly using recombinant components, remaining a vital issue that the relevance in vivo are unknown. Moreover, different MARTs show diverse patterns of tissue distribution, and might possess relevant biofunctions in specific organizations. Thence, more experiments in cells and animals are urgently in need to confirm previous hypothesis and provide better understanding biological influences of MARylation.

### To develop specific MARylation detection methods

Although significant progress is made in developing techniques for ADP-ribosylated targets detection and analysis, it is still not enough, especially for MARylated molecules. Given the biological importance of MARylation is beginning to emerge, further development and refinement of MAR-specific methods will provide a tremendous progress in the field. Reliable tools will contribute to identify and functional assessment of specific sites of MARylated substrates, as well as advance the understanding of MARylation’s functions and mechanisms.

### To pay attention on nucleic acids MARylation

Unlike protein, nucleic acid MARylation is newly identified and in a more emerging field that leaving some objectives of further research about MAR-RNA and MAR-DNA to achieve. One is to further confirm the existence of MARylation on nucleic acids, in particular RNA, in vivo. The other one is to reveal the regulatory mechanisms of MARylation-associated enzymes to understand their substrates and substrate recognition pattern. In addition, it is required to comprehensively elaborate the outcomes of MARylation on nucleic acids and their downstream events. Better addressing all the remaining problems will enormously advance the deeply understanding of MARylation in response to physiological and pathological signals.

### To identify undiscovered enzymes regulating MARylation reactions

Following rapid advances in the MARylation field, increasing ARTs and hydrolyses members have been identified. However, we are still far from having a clear image of MARyaltion system and there are probably remaining undiscovered enzymes modulating MARylation reactions. Identification of novel enzymes possessing a substrate MARylation activity will contribute to understanding of this extraordinary modification and also provide potential targets of specific inhibitors for human therapy. Remarkably, to find out the human homologs of MARylation-relating enzymes in bacteria, fungi or viruses may be an effective strategy. For instance, TRPT1 family was initially found in *Saccharomyces cerevisiae* [195] and its human version referred to as TRPT1. Furthermore, it is also a feasible method to explore the MARylation activity from those known enzymes.

### To reveal the potential connection between MARylation and PARylation

Growing evidence support that MARylation has close association with PARylation. As above, an established PAR transferase PAPR1 is a target of Sirt6 [15]. Under oxidative stress, Sirt6 activates PAPR1 in MARylation-dependent manner to regulate DNA repair pathway [15]. In addition, MARylation functions as a second wave of PARP1 pathway via recruitment of a MARyationn reader RNF114 in response to DNA damage [177]. Besides genomic stability, PARP1 play roles in various cellular processes thus this co-modulatory effect mediated by MARylation and PARylation may exists in diverse pathophysiology conditions. Moreover, with improvement of detection techniques, we may uncover more biological significances and mechanisms behind the crosslink between MAR and PAR.

## Data Availability

All data are available in the manuscript; expression constructs are available on request.
